# 1,1,2,2-Tetra­kis(dimethyl­amino)­ethane-1,2-diium bis­(tetra­phenyl­borate) acetone disolvate

**DOI:** 10.1107/S1600536812015085

**Published:** 2012-04-13

**Authors:** Ioannis Tiritiris, Willi Kantlehner

**Affiliations:** a Institut für Organische Chemie, Universität Stuttgart, Pfaffenwaldring 55, 70569 Stuttgart, Germany; b Fakultät Chemie/Organische Chemie, Hochschule Aalen, Beethovenstrasse 1, D-73430 Aalen, Germany

## Abstract

The title compound, C_10_H_24_N_4_^2+^·2C_24_H_20_B^−^·2C_3_H_6_O, crystallizes with two acetone solvent mol­ecules per asymmetric unit. In the dication, both amidinium units are twisted about the central C—C single bond by 63.8 (3)° and the positive charges are delocalized over both N—C—N planes.

## Related literature

For the crystal structure of tetra­kis­(dimethyl­amino)­ethyl­ene (TDAE), see: Bock *et al.* (1991 ▶). For the synthesis of octa­methyl­oxamidinium salts with different anions, see: Wiberg (1968 ▶). For the synthesis and crystal structures of (TDAE)Cl_2_ and (TDAE)Br_2_, see: Bock *et al.* (1989 ▶). For the synthesis and crystal structure of (TDAE)(SCF_3_)_2_, see: Kolomeitsev *et al.* (2000 ▶). For the crystal structure of (TDAE)(PF_6_)_2_, see: Elbl-Weiser *et al.* (1990 ▶). For the synthesis and crystal structure of *N*,*N*,*N*′,*N*′,*N*′′,*N*′′,*N*′′′,*N*′′′-octa­methyl-(but-2-yne) bis­(amidin­ium) bis­(tetra­fluoro­borate), see: Drandarov *et al.* (2012 ▶). For *N*,*N*,*N*′,*N*′,*N*′′,*N*′′,*N*′′′,*N*′′′-octa­methyl-(but-2-yne) bis­(amidin­ium) bis­(tetra­phenyl­borate), see: Kress *et al.* (2012 ▶).
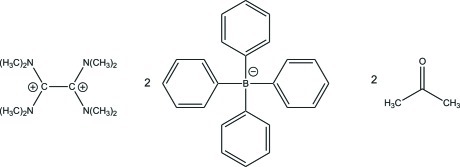


## Experimental

### 

#### Crystal data

C_10_H_24_N_4_^2+^·2C_24_H_20_B^−^·2C_3_H_6_O
*M* *_r_* = 954.91
Orthorhombic, 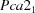

*a* = 30.0601 (9) Å
*b* = 9.9321 (3) Å
*c* = 18.2054 (6) Å
*V* = 5435.4 (3) Å^3^
*Z* = 4
Mo *K*α radiation
μ = 0.07 mm^−1^
*T* = 100 K
0.30 × 0.24 × 0.18 mm

#### Data collection

Bruker Kappa APEXII DUO diffractometer
36726 measured reflections
4983 independent reflections
4058 reflections with *I* > 2σ(*I*)
*R*_int_ = 0.054

#### Refinement

*R*[*F* ^2^ > 2σ(*F* ^2^)] = 0.046
*wR*(*F* ^2^) = 0.109
*S* = 1.05
4983 reflections
661 parameters
1 restraint
H-atom parameters constrained
Δρ_max_ = 0.39 e Å^−3^
Δρ_min_ = −0.30 e Å^−3^

### 

Data collection: *APEX2*, (Bruker, 2008 ▶); cell refinement: *SAINT* (Bruker, 2008 ▶); data reduction: *SAINT*; program(s) used to solve structure: *SHELXS97* (Sheldrick, 2008 ▶); program(s) used to refine structure: *SHELXL97*; molecular graphics: *DIAMOND* (Brandenburg & Putz, 2005 ▶); software used to prepare material for publication: *SHELXL97*.

## Supplementary Material

Crystal structure: contains datablock(s) I, global. DOI: 10.1107/S1600536812015085/fk2058sup1.cif

Structure factors: contains datablock(s) I. DOI: 10.1107/S1600536812015085/fk2058Isup2.hkl

Additional supplementary materials:  crystallographic information; 3D view; checkCIF report

## Figures and Tables

**Fig. 1.**
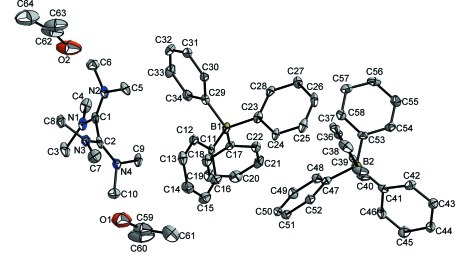
Molecular structure of the title compound showing 50% probability displacement ellipsoids. All H atoms were omitted for clarity.

## supplementary crystallographic information

### Comment

By oxidizing tetrakis(dimethylamino)ethylene (TDAE) with elemental halogens in organic solvents, the octamethyloxamidinium ion (TDAE)^2+^ is formed (Wiberg, 1968). This is also associated with major structural changes. In the here studied bis(tetraphenylborate) salt (Fig. 1), the single bond between C1 and C2 is clearly characterized by a length of 1.498 (5) Å and the C–N bond lengths are equal with 1.309 (4) to 1.330 (4) Å, indicating double bond character. The bonds between the N atoms and the terminal *C*-methyl groups, all have values close to a typical single bond (1.463 (5)–1.479 (5) Å). The sums of angles around the central C1, C2 and the N atoms are all close to 360°. As a consequence of the two-electron oxidation, the presence of four planar dimethylamino groups and a nearly perfect delocalization of each positive charge in the amidinium systems is observed. The charges are distributed in the dication between the dimethylamino groups. The planes built up from both amidinium units (N1/C1/N2 and N3/C2/N4) are twisted to each other by 63.8 (3)°. Similar arrangements have been observed in the crystal structures of (TDAE)Cl_2_ (Bock *et al.*, 1989), (TDAE)Br_2_ (Bock *et al.*, 1989), (TDAE)(SCF_3_)_2_ (Kolomeitsev *et al.*, 2000) and (TDAE)(PF_6_)_2_ (Elbl-Weiser *et al.*, 1990). In contrast to the previously mentioned salts, which are all colourless, the title compound is yellow coloured. The same phenomenon was observed when the colours of *N*,*N*,*N*',*N*',*N*'',*N*'', *N*''',*N*'''-octamethyl-(but-2-yne)-bis(amidinium)- bis(tetrafluoroborate) (Drandarov *et al.*, 2012) and -bis(tetraphenylborate) (Kress *et al.*, 2012) were compared.

### Experimental

The title compound was prepared by reaction of sodium tetraphenylborate with *N*,*N*,*N*',*N*',*N*'',*N*'',*N*''', *N*'''-octamethyloxamidinium dichloride in acetonitrile at room temperature. Yellow single crystals were obtained by slow evaporation of an acetone solution at room temperature.

### Refinement

The title compound crystallizes in the non-centrosymmetric space group Pca2_1_; however, in the absence of significant anomalous scattering effects, the Flack parameter is essentially meaningless. Accordingly, Friedel pairs were merged. Hydrogen atoms bound to aromatic carbon atoms were placed in idealized positions with d(C—H) = 0.95 Å and were included in the refinement in the riding model approximation with *U*(H) set to 1.2 *U*_eq_(C). The hydrogen atoms of the methyl groups were allowed to rotate with a fixed angle around the C–N bond to best fit the experimental electron density, with *U*(H) set to 1.5 *U*_eq_(C) and d(C—H) = 0.98 Å.

### Crystal data

**Table:**

C_10_H_24_N_4_^2+^·2C_24_H_20_B^−^·2C_3_H_6_O	*D*_x_ = 1.167 Mg m^−^^3^
*M**_r_* = 954.91	Melting point: 483 K
Orthorhombic, *P**c**a*2_1_	Mo *K*α radiation, λ = 0.71073 Å
Hall symbol: P 2c -2ac	Cell parameters from 9611 reflections
*a* = 30.0601 (9) Å	θ = 1.4–25.1°
*b* = 9.9321 (3) Å	µ = 0.07 mm^−^^1^
*c* = 18.2054 (6) Å	*T* = 100 K
*V* = 5435.4 (3) Å^3^	Block, yellow
*Z* = 4	0.30 × 0.24 × 0.18 mm
*F*(000) = 2056	

### Data collection

**Table:**

Bruker Kappa APEXII DUO diffractometer	4058 reflections with *I* > 2σ(*I*)
Radiation source: sealed tube	*R*_int_ = 0.054
Graphite monochromator	θ_max_ = 25.1°, θ_min_ = 1.4°
φ scans, and ω scans	*h* = −35→34
36726 measured reflections	*k* = −11→11
4983 independent reflections	*l* = −21→21

### Refinement

**Table:**

Refinement on *F*^2^	Primary atom site location: structure-invariant direct methods
Least-squares matrix: full	Secondary atom site location: difference Fourier map
*R*[*F*^2^ > 2σ(*F*^2^)] = 0.046	Hydrogen site location: difference Fourier map
*wR*(*F*^2^) = 0.109	H-atom parameters constrained
*S* = 1.05	*w* = 1/[σ^2^(*F*_o_^2^) + (0.0601*P*)^2^ + 0.9312*P*] where *P* = (*F*_o_^2^ + 2*F*_c_^2^)/3
4983 reflections	(Δ/σ)_max_ < 0.001
661 parameters	Δρ_max_ = 0.39 e Å^−^^3^
1 restraint	Δρ_min_ = −0.30 e Å^−^^3^

### Special details

**Table:**

Geometry. All e.s.d.'s (except the e.s.d. in the dihedral angle between two l.s. planes) are estimated using the full covariance matrix. The cell e.s.d.'s are taken into account individually in the estimation of e.s.d.'s in distances, angles and torsion angles; correlations between e.s.d.'s in cell parameters are only used when they are defined by crystal symmetry. An approximate (isotropic) treatment of cell e.s.d.'s is used for estimating e.s.d.'s involving l.s. planes.
Refinement. Refinement of *F*^2^ against ALL reflections. The weighted *R*-factor *wR* and goodness of fit *S* are based on *F*^2^, conventional *R*-factors *R* are based on *F*, with *F* set to zero for negative *F*^2^. The threshold expression of *F*^2^ > σ(*F*^2^) is used only for calculating *R*-factors(gt) *etc*. and is not relevant to the choice of reflections for refinement. *R*-factors based on *F*^2^ are statistically about twice as large as those based on *F*, and *R*- factors based on ALL data will be even larger.

### Fractional atomic coordinates and isotropic or equivalent isotropic displacement parameters (Å^2^)

**Table:**

	*x*	*y*	*z*	*U*_iso_*/*U*_eq_	
C1	0.12707 (10)	0.7168 (4)	0.37772 (19)	0.0200 (8)	
C2	0.13799 (11)	0.7692 (3)	0.3027 (2)	0.0220 (8)	
N1	0.14503 (9)	0.6019 (3)	0.39743 (16)	0.0233 (7)	
C3	0.16208 (12)	0.5055 (4)	0.3436 (2)	0.0338 (9)	
H3A	0.1542	0.5355	0.2940	0.051*	
H3B	0.1489	0.4168	0.3528	0.051*	
H3C	0.1945	0.4995	0.3480	0.051*	
C4	0.15054 (12)	0.5609 (4)	0.4744 (2)	0.0320 (9)	
H4A	0.1484	0.6403	0.5062	0.048*	
H4B	0.1797	0.5187	0.4809	0.048*	
H4C	0.1272	0.4965	0.4876	0.048*	
N2	0.10013 (9)	0.7912 (3)	0.41826 (16)	0.0228 (7)	
C5	0.09189 (12)	0.9346 (4)	0.4024 (2)	0.0282 (9)	
H5A	0.1146	0.9679	0.3682	0.042*	
H5B	0.0933	0.9864	0.4482	0.042*	
H5C	0.0624	0.9449	0.3802	0.042*	
C6	0.07184 (12)	0.7379 (4)	0.4777 (2)	0.0312 (9)	
H6A	0.0702	0.6396	0.4737	0.047*	
H6B	0.0419	0.7761	0.4734	0.047*	
H6C	0.0846	0.7625	0.5253	0.047*	
N3	0.10484 (9)	0.7788 (3)	0.25641 (16)	0.0232 (7)	
C7	0.10512 (12)	0.8684 (4)	0.1915 (2)	0.0344 (9)	
H7A	0.1288	0.9354	0.1969	0.052*	
H7B	0.0764	0.9143	0.1875	0.052*	
H7C	0.1104	0.8149	0.1471	0.052*	
C8	0.06283 (11)	0.7054 (4)	0.2677 (2)	0.0281 (9)	
H8A	0.0675	0.6329	0.3034	0.042*	
H8B	0.0529	0.6668	0.2209	0.042*	
H8C	0.0401	0.7675	0.2863	0.042*	
N4	0.18007 (9)	0.8008 (3)	0.28813 (16)	0.0232 (7)	
C9	0.21206 (11)	0.8266 (4)	0.3481 (2)	0.0272 (8)	
H9A	0.1958	0.8462	0.3935	0.041*	
H9B	0.2308	0.9039	0.3353	0.041*	
H9C	0.2308	0.7470	0.3554	0.041*	
C10	0.19960 (12)	0.7968 (4)	0.2144 (2)	0.0322 (9)	
H10A	0.1805	0.7436	0.1819	0.048*	
H10B	0.2292	0.7556	0.2168	0.048*	
H10C	0.2022	0.8887	0.1952	0.048*	
B1	0.24717 (12)	0.1824 (4)	0.4752 (2)	0.0188 (8)	
C11	0.23805 (10)	0.1708 (3)	0.38587 (19)	0.0181 (7)	
C12	0.19512 (11)	0.1679 (3)	0.35475 (19)	0.0223 (8)	
H12A	0.1701	0.1690	0.3865	0.027*	
C13	0.18807 (12)	0.1636 (4)	0.2796 (2)	0.0265 (8)	
H13A	0.1585	0.1630	0.2610	0.032*	
C14	0.22340 (13)	0.1601 (4)	0.2316 (2)	0.0311 (9)	
H14A	0.2186	0.1557	0.1801	0.037*	
C15	0.26608 (13)	0.1631 (4)	0.2598 (2)	0.0323 (9)	
H15A	0.2909	0.1616	0.2275	0.039*	
C16	0.27273 (11)	0.1682 (4)	0.3351 (2)	0.0255 (8)	
H16A	0.3024	0.1701	0.3529	0.031*	
C17	0.29451 (10)	0.1072 (3)	0.49522 (19)	0.0189 (7)	
C18	0.30372 (11)	−0.0243 (4)	0.4717 (2)	0.0243 (8)	
H18A	0.2818	−0.0705	0.4438	0.029*	
C19	0.34317 (11)	−0.0900 (4)	0.4872 (2)	0.0287 (9)	
H19A	0.3477	−0.1796	0.4705	0.034*	
C20	0.37621 (11)	−0.0253 (4)	0.5272 (2)	0.0287 (9)	
H20A	0.4036	−0.0690	0.5373	0.034*	
C21	0.36841 (11)	0.1035 (4)	0.5519 (2)	0.0278 (9)	
H21A	0.3905	0.1487	0.5799	0.033*	
C22	0.32844 (11)	0.1680 (4)	0.53598 (19)	0.0227 (8)	
H22A	0.3240	0.2570	0.5536	0.027*	
C23	0.25020 (10)	0.3409 (3)	0.50010 (19)	0.0188 (7)	
C24	0.26607 (10)	0.4427 (3)	0.45320 (19)	0.0201 (8)	
H24A	0.2737	0.4204	0.4040	0.024*	
C25	0.27097 (11)	0.5748 (4)	0.4767 (2)	0.0281 (9)	
H25A	0.2817	0.6410	0.4434	0.034*	
C26	0.26053 (11)	0.6112 (4)	0.5475 (2)	0.0272 (9)	
H26A	0.2641	0.7018	0.5631	0.033*	
C27	0.24495 (11)	0.5157 (3)	0.5953 (2)	0.0230 (8)	
H27A	0.2374	0.5398	0.6443	0.028*	
C28	0.24023 (10)	0.3832 (3)	0.57183 (19)	0.0196 (8)	
H28A	0.2298	0.3182	0.6060	0.024*	
C29	0.20597 (11)	0.1089 (3)	0.52084 (18)	0.0190 (8)	
C30	0.16441 (11)	0.1728 (4)	0.52823 (19)	0.0210 (8)	
H30A	0.1605	0.2590	0.5065	0.025*	
C31	0.12900 (11)	0.1158 (4)	0.56579 (19)	0.0256 (8)	
H31A	0.1017	0.1634	0.5698	0.031*	
C32	0.13319 (12)	−0.0089 (4)	0.5972 (2)	0.0315 (9)	
H32A	0.1089	−0.0492	0.6224	0.038*	
C33	0.17346 (13)	−0.0747 (4)	0.5914 (2)	0.0357 (10)	
H33A	0.1770	−0.1607	0.6134	0.043*	
C34	0.20880 (12)	−0.0171 (4)	0.5539 (2)	0.0271 (8)	
H34A	0.2360	−0.0655	0.5507	0.033*	
B2	0.00931 (12)	0.3446 (4)	0.2005 (2)	0.0188 (8)	
C35	0.04912 (11)	0.4242 (4)	0.15515 (18)	0.0194 (7)	
C36	0.09428 (10)	0.4095 (3)	0.17347 (19)	0.0217 (8)	
H36A	0.1021	0.3535	0.2137	0.026*	
C37	0.12799 (12)	0.4743 (4)	0.1345 (2)	0.0283 (9)	
H37A	0.1581	0.4609	0.1484	0.034*	
C38	0.11829 (12)	0.5568 (4)	0.0767 (2)	0.0332 (10)	
H38A	0.1414	0.6017	0.0509	0.040*	
C39	0.07416 (13)	0.5741 (5)	0.0562 (2)	0.0363 (10)	
H39A	0.0668	0.6310	0.0161	0.044*	
C40	0.04072 (11)	0.5075 (4)	0.0948 (2)	0.0264 (8)	
H40A	0.0108	0.5193	0.0793	0.032*	
C41	−0.03901 (10)	0.4195 (3)	0.18651 (18)	0.0189 (7)	
C42	−0.04449 (11)	0.5592 (4)	0.19129 (19)	0.0231 (8)	
H42A	−0.0191	0.6126	0.2020	0.028*	
C43	−0.08479 (11)	0.6238 (4)	0.18133 (19)	0.0252 (8)	
H43A	−0.0865	0.7188	0.1867	0.030*	
C44	−0.12205 (12)	0.5531 (4)	0.1640 (2)	0.0336 (10)	
H44A	−0.1499	0.5971	0.1580	0.040*	
C45	−0.11844 (12)	0.4168 (4)	0.1554 (2)	0.0363 (10)	
H45A	−0.1437	0.3661	0.1409	0.044*	
C46	−0.07803 (11)	0.3514 (4)	0.1677 (2)	0.0285 (9)	
H46A	−0.0770	0.2561	0.1630	0.034*	
C47	0.00597 (10)	0.1890 (4)	0.17045 (19)	0.0205 (8)	
C48	−0.00876 (11)	0.0813 (4)	0.2141 (2)	0.0240 (8)	
H48A	−0.0142	0.0971	0.2648	0.029*	
C49	−0.01574 (11)	−0.0472 (4)	0.1867 (2)	0.0285 (9)	
H49A	−0.0259	−0.1164	0.2185	0.034*	
C50	−0.00809 (11)	−0.0752 (4)	0.1140 (2)	0.0271 (9)	
H50A	−0.0128	−0.1633	0.0953	0.032*	
C51	0.00657 (11)	0.0272 (4)	0.0681 (2)	0.0274 (9)	
H51A	0.0118	0.0099	0.0176	0.033*	
C52	0.01353 (11)	0.1557 (4)	0.0967 (2)	0.0235 (8)	
H52A	0.0239	0.2242	0.0645	0.028*	
C53	0.02121 (10)	0.3463 (3)	0.28940 (19)	0.0187 (7)	
C54	0.00099 (10)	0.4337 (4)	0.33938 (19)	0.0209 (7)	
H54A	−0.0218	0.4919	0.3220	0.025*	
C55	0.01254 (11)	0.4395 (4)	0.41325 (19)	0.0262 (8)	
H55A	−0.0023	0.5007	0.4450	0.031*	
C56	0.04521 (12)	0.3573 (4)	0.4405 (2)	0.0279 (9)	
H56A	0.0535	0.3617	0.4908	0.033*	
C57	0.06587 (11)	0.2681 (4)	0.3933 (2)	0.0267 (9)	
H57A	0.0886	0.2104	0.4113	0.032*	
C58	0.05373 (11)	0.2622 (4)	0.3203 (2)	0.0239 (8)	
H58A	0.0680	0.1982	0.2896	0.029*	
O1	0.29178 (12)	0.6328 (4)	0.2820 (2)	0.0693 (11)	
C59	0.32840 (16)	0.6770 (6)	0.3005 (3)	0.0565 (14)	
C60	0.36004 (18)	0.5888 (8)	0.3382 (3)	0.086 (2)	
H60A	0.3681	0.6285	0.3857	0.129*	
H60B	0.3868	0.5784	0.3080	0.129*	
H60C	0.3464	0.5004	0.3462	0.129*	
C61	0.3405 (2)	0.8204 (6)	0.2871 (3)	0.0740 (17)	
H61A	0.3174	0.8631	0.2570	0.111*	
H61B	0.3690	0.8246	0.2613	0.111*	
H61C	0.3429	0.8678	0.3341	0.111*	
O2	0.47849 (12)	0.1730 (5)	0.3833 (2)	0.0902 (15)	
C62	0.43772 (18)	0.1911 (7)	0.3802 (3)	0.0662 (17)	
C63	0.40778 (16)	0.0963 (7)	0.3460 (3)	0.0734 (17)	
H63A	0.3938	0.1382	0.3030	0.110*	
H63B	0.3847	0.0699	0.3812	0.110*	
H63C	0.4245	0.0164	0.3305	0.110*	
C64	0.4158 (2)	0.3130 (7)	0.4088 (4)	0.095 (2)	
H64A	0.4359	0.3594	0.4428	0.142*	
H64B	0.3885	0.2875	0.4348	0.142*	
H64C	0.4083	0.3731	0.3679	0.142*	

### Atomic displacement parameters (Å^2^)

**Table:**

	*U*^11^	*U*^22^	*U*^33^	*U*^12^	*U*^13^	*U*^23^
C1	0.0152 (16)	0.0201 (19)	0.0246 (19)	0.0010 (14)	−0.0072 (14)	−0.0050 (16)
C2	0.0197 (18)	0.0111 (18)	0.035 (2)	0.0040 (14)	−0.0055 (16)	−0.0089 (16)
N1	0.0204 (14)	0.0149 (16)	0.0347 (17)	0.0001 (12)	−0.0083 (13)	−0.0015 (13)
C3	0.030 (2)	0.016 (2)	0.055 (3)	0.0080 (16)	−0.0070 (19)	−0.0104 (19)
C4	0.027 (2)	0.026 (2)	0.043 (2)	−0.0009 (16)	−0.0094 (17)	0.0097 (19)
N2	0.0216 (15)	0.0168 (16)	0.0300 (16)	0.0025 (13)	−0.0047 (13)	−0.0031 (13)
C5	0.035 (2)	0.019 (2)	0.031 (2)	0.0076 (16)	−0.0051 (16)	−0.0051 (17)
C6	0.0277 (19)	0.031 (2)	0.035 (2)	0.0048 (16)	0.0025 (17)	0.0003 (19)
N3	0.0193 (14)	0.0223 (16)	0.0282 (16)	−0.0001 (12)	−0.0036 (13)	−0.0049 (14)
C7	0.032 (2)	0.042 (3)	0.029 (2)	0.0001 (18)	−0.0065 (17)	0.0055 (19)
C8	0.0207 (18)	0.028 (2)	0.035 (2)	−0.0031 (16)	−0.0084 (16)	−0.0093 (18)
N4	0.0199 (15)	0.0192 (17)	0.0306 (16)	0.0010 (12)	−0.0024 (13)	−0.0073 (13)
C9	0.0206 (18)	0.022 (2)	0.039 (2)	−0.0028 (15)	−0.0083 (16)	−0.0030 (17)
C10	0.027 (2)	0.034 (2)	0.035 (2)	0.0010 (17)	0.0041 (17)	−0.0056 (19)
B1	0.0169 (18)	0.015 (2)	0.025 (2)	−0.0009 (15)	−0.0046 (16)	0.0021 (17)
C11	0.0207 (17)	0.0089 (17)	0.0248 (18)	−0.0004 (13)	−0.0012 (15)	−0.0002 (14)
C12	0.0196 (17)	0.0193 (19)	0.028 (2)	−0.0002 (14)	−0.0004 (15)	−0.0022 (16)
C13	0.0265 (19)	0.020 (2)	0.033 (2)	0.0016 (16)	−0.0108 (16)	−0.0010 (17)
C14	0.038 (2)	0.033 (2)	0.022 (2)	−0.0005 (18)	−0.0036 (17)	0.0027 (18)
C15	0.030 (2)	0.037 (2)	0.029 (2)	−0.0027 (17)	0.0039 (17)	0.0041 (19)
C16	0.0194 (18)	0.029 (2)	0.028 (2)	−0.0006 (15)	0.0003 (15)	0.0033 (17)
C17	0.0150 (16)	0.0188 (19)	0.0228 (18)	−0.0033 (14)	0.0021 (14)	0.0038 (15)
C18	0.0214 (18)	0.023 (2)	0.029 (2)	0.0013 (15)	−0.0052 (15)	0.0000 (16)
C19	0.0245 (19)	0.023 (2)	0.039 (2)	0.0040 (16)	−0.0009 (17)	0.0036 (18)
C20	0.022 (2)	0.030 (2)	0.034 (2)	0.0072 (16)	−0.0030 (16)	0.0051 (18)
C21	0.0188 (18)	0.033 (2)	0.031 (2)	−0.0051 (16)	−0.0072 (16)	0.0042 (18)
C22	0.0207 (18)	0.022 (2)	0.0257 (19)	−0.0035 (15)	−0.0010 (15)	−0.0006 (16)
C23	0.0118 (15)	0.0173 (18)	0.0272 (18)	0.0028 (14)	−0.0069 (14)	0.0038 (16)
C24	0.0147 (16)	0.0180 (19)	0.028 (2)	−0.0034 (14)	−0.0068 (13)	0.0028 (16)
C25	0.0209 (18)	0.021 (2)	0.042 (2)	−0.0023 (15)	−0.0081 (16)	0.0098 (18)
C26	0.0218 (18)	0.0142 (18)	0.046 (3)	−0.0010 (14)	−0.0131 (17)	−0.0070 (18)
C27	0.0212 (18)	0.0185 (19)	0.0293 (19)	0.0016 (15)	−0.0065 (15)	−0.0038 (16)
C28	0.0117 (16)	0.0196 (19)	0.0277 (19)	−0.0009 (13)	−0.0026 (14)	0.0016 (16)
C29	0.0208 (18)	0.0161 (19)	0.0202 (18)	−0.0021 (14)	−0.0028 (14)	−0.0038 (15)
C30	0.0229 (19)	0.0172 (19)	0.0229 (18)	−0.0027 (15)	−0.0007 (14)	−0.0014 (15)
C31	0.0224 (18)	0.028 (2)	0.0259 (19)	−0.0045 (16)	0.0031 (15)	−0.0077 (17)
C32	0.028 (2)	0.031 (2)	0.036 (2)	−0.0136 (17)	0.0097 (17)	−0.0019 (19)
C33	0.046 (2)	0.021 (2)	0.040 (2)	−0.0070 (18)	0.0094 (19)	0.0089 (19)
C34	0.029 (2)	0.021 (2)	0.031 (2)	−0.0016 (16)	0.0005 (16)	−0.0029 (17)
B2	0.0157 (18)	0.020 (2)	0.021 (2)	−0.0021 (16)	0.0029 (15)	−0.0019 (17)
C35	0.0190 (17)	0.0212 (19)	0.0180 (17)	−0.0014 (15)	0.0011 (13)	−0.0094 (15)
C36	0.0212 (17)	0.0169 (19)	0.027 (2)	0.0006 (14)	0.0012 (15)	−0.0050 (15)
C37	0.0192 (19)	0.026 (2)	0.040 (2)	−0.0065 (16)	0.0032 (16)	−0.0138 (18)
C38	0.029 (2)	0.042 (2)	0.028 (2)	−0.0171 (18)	0.0109 (17)	−0.0096 (19)
C39	0.040 (2)	0.047 (3)	0.022 (2)	−0.0151 (19)	−0.0016 (17)	0.0033 (19)
C40	0.0188 (18)	0.038 (2)	0.0225 (18)	−0.0091 (16)	−0.0015 (15)	−0.0025 (17)
C41	0.0207 (17)	0.0210 (19)	0.0150 (16)	−0.0036 (14)	0.0010 (13)	−0.0012 (15)
C42	0.0203 (18)	0.023 (2)	0.0265 (19)	−0.0036 (15)	−0.0049 (15)	0.0061 (16)
C43	0.0261 (19)	0.024 (2)	0.0257 (19)	0.0008 (15)	−0.0039 (16)	−0.0013 (16)
C44	0.0225 (19)	0.032 (2)	0.046 (2)	0.0067 (17)	−0.0054 (17)	−0.015 (2)
C45	0.0199 (19)	0.035 (3)	0.054 (3)	−0.0042 (17)	−0.0044 (18)	−0.010 (2)
C46	0.0205 (18)	0.021 (2)	0.044 (2)	−0.0026 (15)	0.0015 (16)	−0.0107 (18)
C47	0.0119 (15)	0.025 (2)	0.024 (2)	−0.0004 (14)	−0.0023 (14)	−0.0018 (16)
C48	0.0212 (18)	0.022 (2)	0.029 (2)	−0.0005 (15)	−0.0018 (16)	−0.0002 (17)
C49	0.0270 (19)	0.019 (2)	0.039 (2)	−0.0025 (15)	−0.0063 (17)	0.0016 (18)
C50	0.0185 (17)	0.017 (2)	0.045 (2)	0.0021 (15)	−0.0103 (16)	−0.0091 (18)
C51	0.0159 (18)	0.029 (2)	0.038 (2)	0.0014 (15)	−0.0042 (16)	−0.0105 (19)
C52	0.0161 (17)	0.024 (2)	0.030 (2)	−0.0013 (14)	0.0001 (15)	−0.0029 (17)
C53	0.0160 (16)	0.0173 (18)	0.0229 (18)	−0.0059 (14)	−0.0033 (14)	−0.0005 (15)
C54	0.0182 (17)	0.0240 (19)	0.0204 (17)	−0.0016 (14)	0.0008 (14)	0.0010 (16)
C55	0.0270 (19)	0.030 (2)	0.0218 (19)	0.0001 (16)	0.0038 (15)	−0.0010 (17)
C56	0.033 (2)	0.032 (2)	0.0187 (18)	−0.0072 (17)	−0.0076 (16)	0.0045 (17)
C57	0.0240 (19)	0.026 (2)	0.030 (2)	−0.0032 (16)	−0.0096 (16)	0.0062 (18)
C58	0.0220 (18)	0.0193 (19)	0.031 (2)	−0.0014 (15)	−0.0002 (15)	0.0001 (16)
O1	0.051 (2)	0.088 (3)	0.069 (2)	0.011 (2)	0.0060 (18)	−0.008 (2)
C59	0.043 (3)	0.081 (4)	0.046 (3)	0.010 (3)	0.003 (2)	−0.003 (3)
C60	0.067 (4)	0.141 (6)	0.050 (3)	0.032 (4)	0.008 (3)	0.015 (4)
C61	0.082 (4)	0.082 (4)	0.059 (3)	−0.010 (3)	0.008 (3)	−0.022 (3)
O2	0.046 (2)	0.165 (5)	0.060 (2)	−0.028 (2)	−0.0040 (19)	0.018 (3)
C62	0.053 (3)	0.109 (5)	0.037 (3)	−0.025 (3)	0.012 (2)	0.011 (3)
C63	0.047 (3)	0.112 (5)	0.061 (3)	−0.023 (3)	0.018 (3)	−0.006 (4)
C64	0.073 (4)	0.105 (6)	0.106 (6)	−0.026 (4)	0.031 (4)	0.000 (5)

### Geometric parameters (Å, º)

**Table:**

C1—N1	1.313 (4)	C30—C31	1.386 (5)
C1—N2	1.321 (4)	C30—H30A	0.9500
C1—C2	1.498 (5)	C31—C32	1.370 (5)
C2—N3	1.309 (4)	C31—H31A	0.9500
C2—N4	1.330 (4)	C32—C33	1.380 (6)
N1—C3	1.463 (5)	C32—H32A	0.9500
N1—C4	1.469 (5)	C33—C34	1.386 (5)
C3—H3A	0.9800	C33—H33A	0.9500
C3—H3B	0.9800	C34—H34A	0.9500
C3—H3C	0.9800	B2—C47	1.642 (5)
C4—H4A	0.9800	B2—C41	1.652 (5)
C4—H4B	0.9800	B2—C35	1.655 (5)
C4—H4C	0.9800	B2—C53	1.658 (5)
N2—C6	1.474 (5)	C35—C40	1.399 (5)
N2—C5	1.475 (5)	C35—C36	1.405 (5)
C5—H5A	0.9800	C36—C37	1.394 (5)
C5—H5B	0.9800	C36—H36A	0.9500
C5—H5C	0.9800	C37—C38	1.365 (6)
C6—H6A	0.9800	C37—H37A	0.9500
C6—H6B	0.9800	C38—C39	1.389 (5)
C6—H6C	0.9800	C38—H38A	0.9500
N3—C8	1.473 (4)	C39—C40	1.393 (5)
N3—C7	1.479 (5)	C39—H39A	0.9500
C7—H7A	0.9800	C40—H40A	0.9500
C7—H7B	0.9800	C41—C46	1.397 (5)
C7—H7C	0.9800	C41—C42	1.400 (5)
C8—H8A	0.9800	C42—C43	1.383 (5)
C8—H8B	0.9800	C42—H42A	0.9500
C8—H8C	0.9800	C43—C44	1.360 (5)
N4—C10	1.465 (5)	C43—H43A	0.9500
N4—C9	1.478 (4)	C44—C45	1.366 (5)
C9—H9A	0.9800	C44—H44A	0.9500
C9—H9B	0.9800	C45—C46	1.396 (5)
C9—H9C	0.9800	C45—H45A	0.9500
C10—H10A	0.9800	C46—H46A	0.9500
C10—H10B	0.9800	C47—C52	1.401 (5)
C10—H10C	0.9800	C47—C48	1.405 (5)
B1—C23	1.641 (5)	C48—C49	1.386 (5)
B1—C17	1.648 (5)	C48—H48A	0.9500
B1—C11	1.654 (5)	C49—C50	1.372 (6)
B1—C29	1.660 (5)	C49—H49A	0.9500
C11—C16	1.394 (5)	C50—C51	1.387 (5)
C11—C12	1.410 (5)	C50—H50A	0.9500
C12—C13	1.385 (5)	C51—C52	1.394 (5)
C12—H12A	0.9500	C51—H51A	0.9500
C13—C14	1.376 (5)	C52—H52A	0.9500
C13—H13A	0.9500	C53—C54	1.397 (5)
C14—C15	1.382 (5)	C53—C58	1.404 (5)
C14—H14A	0.9500	C54—C55	1.390 (5)
C15—C16	1.385 (5)	C54—H54A	0.9500
C15—H15A	0.9500	C55—C56	1.370 (5)
C16—H16A	0.9500	C55—H55A	0.9500
C17—C22	1.398 (5)	C56—C57	1.381 (5)
C17—C18	1.402 (5)	C56—H56A	0.9500
C18—C19	1.383 (5)	C57—C58	1.379 (5)
C18—H18A	0.9500	C57—H57A	0.9500
C19—C20	1.389 (5)	C58—H58A	0.9500
C19—H19A	0.9500	O1—C59	1.232 (6)
C20—C21	1.375 (5)	C59—C60	1.464 (7)
C20—H20A	0.9500	C59—C61	1.490 (8)
C21—C22	1.392 (5)	C60—H60A	0.9800
C21—H21A	0.9500	C60—H60B	0.9800
C22—H22A	0.9500	C60—H60C	0.9800
C23—C28	1.404 (5)	C61—H61A	0.9800
C23—C24	1.407 (5)	C61—H61B	0.9800
C24—C25	1.388 (5)	C61—H61C	0.9800
C24—H24A	0.9500	O2—C62	1.240 (6)
C25—C26	1.374 (6)	C62—C63	1.444 (8)
C25—H25A	0.9500	C62—C64	1.474 (8)
C26—C27	1.371 (5)	C63—H63A	0.9800
C26—H26A	0.9500	C63—H63B	0.9800
C27—C28	1.391 (5)	C63—H63C	0.9800
C27—H27A	0.9500	C64—H64A	0.9800
C28—H28A	0.9500	C64—H64B	0.9800
C29—C34	1.391 (5)	C64—H64C	0.9800
C29—C30	1.408 (5)		
			
N1—C1—N2	125.8 (3)	C34—C29—C30	114.7 (3)
N1—C1—C2	117.5 (3)	C34—C29—B1	124.5 (3)
N2—C1—C2	116.7 (3)	C30—C29—B1	120.7 (3)
N3—C2—N4	125.3 (3)	C31—C30—C29	123.0 (3)
N3—C2—C1	116.5 (3)	C31—C30—H30A	118.5
N4—C2—C1	118.2 (3)	C29—C30—H30A	118.5
C1—N1—C3	122.0 (3)	C32—C31—C30	120.3 (3)
C1—N1—C4	123.2 (3)	C32—C31—H31A	119.8
C3—N1—C4	114.7 (3)	C30—C31—H31A	119.8
N1—C3—H3A	109.5	C31—C32—C33	118.5 (3)
N1—C3—H3B	109.5	C31—C32—H32A	120.8
H3A—C3—H3B	109.5	C33—C32—H32A	120.8
N1—C3—H3C	109.5	C32—C33—C34	121.0 (4)
H3A—C3—H3C	109.5	C32—C33—H33A	119.5
H3B—C3—H3C	109.5	C34—C33—H33A	119.5
N1—C4—H4A	109.5	C33—C34—C29	122.5 (3)
N1—C4—H4B	109.5	C33—C34—H34A	118.7
H4A—C4—H4B	109.5	C29—C34—H34A	118.7
N1—C4—H4C	109.5	C47—B2—C41	108.6 (3)
H4A—C4—H4C	109.5	C47—B2—C35	109.1 (3)
H4B—C4—H4C	109.5	C41—B2—C35	110.1 (3)
C1—N2—C6	124.2 (3)	C47—B2—C53	110.4 (3)
C1—N2—C5	122.2 (3)	C41—B2—C53	109.6 (3)
C6—N2—C5	113.2 (3)	C35—B2—C53	109.0 (3)
N2—C5—H5A	109.5	C40—C35—C36	115.0 (3)
N2—C5—H5B	109.5	C40—C35—B2	123.0 (3)
H5A—C5—H5B	109.5	C36—C35—B2	122.0 (3)
N2—C5—H5C	109.5	C37—C36—C35	122.2 (3)
H5A—C5—H5C	109.5	C37—C36—H36A	118.9
H5B—C5—H5C	109.5	C35—C36—H36A	118.9
N2—C6—H6A	109.5	C38—C37—C36	120.9 (3)
N2—C6—H6B	109.5	C38—C37—H37A	119.5
H6A—C6—H6B	109.5	C36—C37—H37A	119.5
N2—C6—H6C	109.5	C37—C38—C39	119.0 (3)
H6A—C6—H6C	109.5	C37—C38—H38A	120.5
H6B—C6—H6C	109.5	C39—C38—H38A	120.5
C2—N3—C8	121.8 (3)	C38—C39—C40	119.7 (4)
C2—N3—C7	123.6 (3)	C38—C39—H39A	120.2
C8—N3—C7	114.5 (3)	C40—C39—H39A	120.2
N3—C7—H7A	109.5	C39—C40—C35	123.1 (3)
N3—C7—H7B	109.5	C39—C40—H40A	118.4
H7A—C7—H7B	109.5	C35—C40—H40A	118.4
N3—C7—H7C	109.5	C46—C41—C42	113.4 (3)
H7A—C7—H7C	109.5	C46—C41—B2	123.9 (3)
H7B—C7—H7C	109.5	C42—C41—B2	122.7 (3)
N3—C8—H8A	109.5	C43—C42—C41	123.7 (3)
N3—C8—H8B	109.5	C43—C42—H42A	118.1
H8A—C8—H8B	109.5	C41—C42—H42A	118.1
N3—C8—H8C	109.5	C44—C43—C42	120.8 (4)
H8A—C8—H8C	109.5	C44—C43—H43A	119.6
H8B—C8—H8C	109.5	C42—C43—H43A	119.6
C2—N4—C10	123.9 (3)	C43—C44—C45	118.2 (4)
C2—N4—C9	120.8 (3)	C43—C44—H44A	120.9
C10—N4—C9	114.9 (3)	C45—C44—H44A	120.9
N4—C9—H9A	109.5	C44—C45—C46	120.8 (4)
N4—C9—H9B	109.5	C44—C45—H45A	119.6
H9A—C9—H9B	109.5	C46—C45—H45A	119.6
N4—C9—H9C	109.5	C45—C46—C41	123.0 (3)
H9A—C9—H9C	109.5	C45—C46—H46A	118.5
H9B—C9—H9C	109.5	C41—C46—H46A	118.5
N4—C10—H10A	109.5	C52—C47—C48	114.4 (3)
N4—C10—H10B	109.5	C52—C47—B2	122.1 (3)
H10A—C10—H10B	109.5	C48—C47—B2	123.2 (3)
N4—C10—H10C	109.5	C49—C48—C47	123.0 (3)
H10A—C10—H10C	109.5	C49—C48—H48A	118.5
H10B—C10—H10C	109.5	C47—C48—H48A	118.5
C23—B1—C17	109.0 (3)	C50—C49—C48	120.6 (4)
C23—B1—C11	110.3 (3)	C50—C49—H49A	119.7
C17—B1—C11	109.2 (3)	C48—C49—H49A	119.7
C23—B1—C29	109.0 (3)	C49—C50—C51	119.0 (3)
C17—B1—C29	109.5 (3)	C49—C50—H50A	120.5
C11—B1—C29	109.7 (3)	C51—C50—H50A	120.5
C16—C11—C12	114.7 (3)	C50—C51—C52	119.6 (4)
C16—C11—B1	122.0 (3)	C50—C51—H51A	120.2
C12—C11—B1	123.3 (3)	C52—C51—H51A	120.2
C13—C12—C11	122.5 (3)	C51—C52—C47	123.3 (4)
C13—C12—H12A	118.7	C51—C52—H52A	118.3
C11—C12—H12A	118.7	C47—C52—H52A	118.3
C14—C13—C12	120.7 (3)	C54—C53—C58	114.3 (3)
C14—C13—H13A	119.7	C54—C53—B2	123.3 (3)
C12—C13—H13A	119.7	C58—C53—B2	122.4 (3)
C13—C14—C15	118.7 (3)	C55—C54—C53	123.2 (3)
C13—C14—H14A	120.7	C55—C54—H54A	118.4
C15—C14—H14A	120.7	C53—C54—H54A	118.4
C14—C15—C16	120.2 (3)	C56—C55—C54	120.3 (3)
C14—C15—H15A	119.9	C56—C55—H55A	119.9
C16—C15—H15A	119.9	C54—C55—H55A	119.9
C15—C16—C11	123.3 (3)	C55—C56—C57	118.7 (3)
C15—C16—H16A	118.4	C55—C56—H56A	120.7
C11—C16—H16A	118.4	C57—C56—H56A	120.7
C22—C17—C18	114.8 (3)	C58—C57—C56	120.5 (3)
C22—C17—B1	123.5 (3)	C58—C57—H57A	119.8
C18—C17—B1	121.7 (3)	C56—C57—H57A	119.8
C19—C18—C17	123.1 (3)	C57—C58—C53	123.0 (3)
C19—C18—H18A	118.4	C57—C58—H58A	118.5
C17—C18—H18A	118.4	C53—C58—H58A	118.5
C18—C19—C20	120.1 (4)	O1—C59—C60	119.7 (6)
C18—C19—H19A	119.9	O1—C59—C61	120.8 (5)
C20—C19—H19A	119.9	C60—C59—C61	119.4 (5)
C21—C20—C19	118.6 (3)	C59—C60—H60A	109.5
C21—C20—H20A	120.7	C59—C60—H60B	109.5
C19—C20—H20A	120.7	H60A—C60—H60B	109.5
C20—C21—C22	120.5 (3)	C59—C60—H60C	109.5
C20—C21—H21A	119.8	H60A—C60—H60C	109.5
C22—C21—H21A	119.8	H60B—C60—H60C	109.5
C21—C22—C17	122.8 (3)	C59—C61—H61A	109.5
C21—C22—H22A	118.6	C59—C61—H61B	109.5
C17—C22—H22A	118.6	H61A—C61—H61B	109.5
C28—C23—C24	114.9 (3)	C59—C61—H61C	109.5
C28—C23—B1	122.1 (3)	H61A—C61—H61C	109.5
C24—C23—B1	122.8 (3)	H61B—C61—H61C	109.5
C25—C24—C23	121.9 (3)	O2—C62—C63	122.8 (6)
C25—C24—H24A	119.0	O2—C62—C64	123.0 (6)
C23—C24—H24A	119.0	C63—C62—C64	114.2 (5)
C26—C25—C24	120.9 (3)	C62—C63—H63A	109.5
C26—C25—H25A	119.6	C62—C63—H63B	109.5
C24—C25—H25A	119.6	H63A—C63—H63B	109.5
C27—C26—C25	119.5 (3)	C62—C63—H63C	109.5
C27—C26—H26A	120.3	H63A—C63—H63C	109.5
C25—C26—H26A	120.3	H63B—C63—H63C	109.5
C26—C27—C28	119.6 (3)	C62—C64—H64A	109.5
C26—C27—H27A	120.2	C62—C64—H64B	109.5
C28—C27—H27A	120.2	H64A—C64—H64B	109.5
C27—C28—C23	123.2 (3)	C62—C64—H64C	109.5
C27—C28—H28A	118.4	H64A—C64—H64C	109.5
C23—C28—H28A	118.4	H64B—C64—H64C	109.5
			
N1—C1—C2—N3	115.9 (3)	C17—B1—C29—C30	164.0 (3)
N2—C1—C2—N3	−64.2 (4)	C11—B1—C29—C30	−76.1 (4)
N1—C1—C2—N4	−63.4 (4)	C34—C29—C30—C31	0.3 (5)
N2—C1—C2—N4	116.4 (3)	B1—C29—C30—C31	−179.9 (3)
N2—C1—N1—C3	159.6 (3)	C29—C30—C31—C32	−0.7 (5)
C2—C1—N1—C3	−20.5 (4)	C30—C31—C32—C33	0.9 (5)
N2—C1—N1—C4	−21.7 (5)	C31—C32—C33—C34	−0.8 (6)
C2—C1—N1—C4	158.1 (3)	C32—C33—C34—C29	0.4 (6)
N1—C1—N2—C6	−25.5 (5)	C30—C29—C34—C33	−0.2 (5)
C2—C1—N2—C6	154.6 (3)	B1—C29—C34—C33	−179.9 (4)
N1—C1—N2—C5	161.5 (3)	C47—B2—C35—C40	97.6 (4)
C2—C1—N2—C5	−18.3 (4)	C41—B2—C35—C40	−21.4 (4)
N4—C2—N3—C8	160.9 (3)	C53—B2—C35—C40	−141.7 (3)
C1—C2—N3—C8	−18.3 (5)	C47—B2—C35—C36	−80.4 (4)
N4—C2—N3—C7	−22.6 (5)	C41—B2—C35—C36	160.5 (3)
C1—C2—N3—C7	158.1 (3)	C53—B2—C35—C36	40.2 (4)
N3—C2—N4—C10	−26.6 (5)	C40—C35—C36—C37	0.5 (5)
C1—C2—N4—C10	152.7 (3)	B2—C35—C36—C37	178.7 (3)
N3—C2—N4—C9	161.1 (3)	C35—C36—C37—C38	0.6 (5)
C1—C2—N4—C9	−19.7 (5)	C36—C37—C38—C39	−1.0 (6)
C23—B1—C11—C16	88.2 (4)	C37—C38—C39—C40	0.1 (6)
C17—B1—C11—C16	−31.6 (4)	C38—C39—C40—C35	1.2 (6)
C29—B1—C11—C16	−151.7 (3)	C36—C35—C40—C39	−1.4 (5)
C23—B1—C11—C12	−89.3 (4)	B2—C35—C40—C39	−179.6 (3)
C17—B1—C11—C12	150.9 (3)	C47—B2—C41—C46	12.9 (5)
C29—B1—C11—C12	30.8 (4)	C35—B2—C41—C46	132.3 (3)
C16—C11—C12—C13	−0.3 (5)	C53—B2—C41—C46	−107.8 (4)
B1—C11—C12—C13	177.3 (3)	C47—B2—C41—C42	−166.0 (3)
C11—C12—C13—C14	0.9 (6)	C35—B2—C41—C42	−46.6 (4)
C12—C13—C14—C15	−1.0 (6)	C53—B2—C41—C42	73.4 (4)
C13—C14—C15—C16	0.6 (6)	C46—C41—C42—C43	2.5 (5)
C14—C15—C16—C11	−0.1 (6)	B2—C41—C42—C43	−178.6 (3)
C12—C11—C16—C15	−0.1 (5)	C41—C42—C43—C44	−1.8 (6)
B1—C11—C16—C15	−177.8 (3)	C42—C43—C44—C45	−1.1 (6)
C23—B1—C17—C22	8.0 (5)	C43—C44—C45—C46	3.1 (6)
C11—B1—C17—C22	128.6 (3)	C44—C45—C46—C41	−2.4 (6)
C29—B1—C17—C22	−111.2 (3)	C42—C41—C46—C45	−0.4 (5)
C23—B1—C17—C18	−171.7 (3)	B2—C41—C46—C45	−179.4 (3)
C11—B1—C17—C18	−51.0 (4)	C41—B2—C47—C52	85.1 (4)
C29—B1—C17—C18	69.1 (4)	C35—B2—C47—C52	−34.9 (4)
C22—C17—C18—C19	0.0 (5)	C53—B2—C47—C52	−154.7 (3)
B1—C17—C18—C19	179.7 (3)	C41—B2—C47—C48	−88.5 (4)
C17—C18—C19—C20	−0.7 (6)	C35—B2—C47—C48	151.5 (3)
C18—C19—C20—C21	1.2 (6)	C53—B2—C47—C48	31.7 (4)
C19—C20—C21—C22	−1.0 (5)	C52—C47—C48—C49	−0.7 (5)
C20—C21—C22—C17	0.2 (5)	B2—C47—C48—C49	173.4 (3)
C18—C17—C22—C21	0.3 (5)	C47—C48—C49—C50	0.4 (5)
B1—C17—C22—C21	−179.4 (3)	C48—C49—C50—C51	−0.3 (5)
C17—B1—C23—C28	−85.8 (4)	C49—C50—C51—C52	0.5 (5)
C11—B1—C23—C28	154.3 (3)	C50—C51—C52—C47	−0.8 (5)
C29—B1—C23—C28	33.7 (4)	C48—C47—C52—C51	0.8 (5)
C17—B1—C23—C24	89.3 (4)	B2—C47—C52—C51	−173.3 (3)
C11—B1—C23—C24	−30.6 (4)	C47—B2—C53—C54	−138.2 (3)
C29—B1—C23—C24	−151.2 (3)	C41—B2—C53—C54	−18.6 (4)
C28—C23—C24—C25	−0.5 (5)	C35—B2—C53—C54	102.0 (3)
B1—C23—C24—C25	−175.9 (3)	C47—B2—C53—C58	43.9 (4)
C23—C24—C25—C26	0.2 (5)	C41—B2—C53—C58	163.5 (3)
C24—C25—C26—C27	−0.2 (5)	C35—B2—C53—C58	−75.9 (4)
C25—C26—C27—C28	0.5 (5)	C58—C53—C54—C55	1.3 (5)
C26—C27—C28—C23	−0.8 (5)	B2—C53—C54—C55	−176.8 (3)
C24—C23—C28—C27	0.8 (5)	C53—C54—C55—C56	0.1 (6)
B1—C23—C28—C27	176.3 (3)	C54—C55—C56—C57	−0.8 (5)
C23—B1—C29—C34	−135.5 (3)	C55—C56—C57—C58	0.0 (5)
C17—B1—C29—C34	−16.3 (5)	C56—C57—C58—C53	1.5 (5)
C11—B1—C29—C34	103.6 (4)	C54—C53—C58—C57	−2.1 (5)
C23—B1—C29—C30	44.8 (4)	B2—C53—C58—C57	176.0 (3)
